# *In Silico* and *In Vivo*: Evaluating the Therapeutic Potential of Kaempferol, Quercetin, and Catechin to Treat Chronic Epilepsy in a Rat Model

**DOI:** 10.3389/fbioe.2021.754952

**Published:** 2021-11-04

**Authors:** Hammad Ahmed, Mahtab Ahmad Khan, Syed Awais Ali Zaidi, Sajjad Muhammad

**Affiliations:** ^1^ Faculty of Pharmacy, The University of Lahore, Defence Road Campus, Lahore, Pakistan; ^2^ Imran Idrees College of Pharmacy, Sialkot, Pakistan; ^3^ Faculty of Pharmacy, University of Central Punjab, Lahore, Pakistan; ^4^ Department of Neurosurgery, Medical Faculty, Heinrich-Heine University of Düsseldorf, Düsseldorf, Germany; ^5^ Department of Neurosurgery, University of Helsinki and University Hospital, Helsinki, Finland

**Keywords:** antiepileptic phytoflavonoids, synaptic vesicle 2A, hippocampus, rat behavior, histopathology of hippocampal sections, molecular docking

## Abstract

Recently, alternative therapies are gaining popularity in the treatment of epilepsy. The present study aimed to find out the antiepileptic potential of quercetin, catechin, and kaempferol. *In vivo* and *in silico* experiments were conducted to investigate their therapeutic potential. 25 mg/kg/day of pentylenetetrazole was administered for 4 weeks after epilepsy was induced in the rats; this was followed by the behavioral studies and histological analysis of rat brain slices. Binding affinities of kaempferol, quercetin, and catechin were assessed by performing *in silico* studies. Kaempferol, quercetin, and catechin were found to have the highest binding affinity with the synaptic vesicle 2A (SV2A) protein, comparable to standard levetiracetam (LEV). The mRNA levels of SV2A, as well as the expression of TNF, IL 6, IL 1 beta, NFkB, IL 1Ra, IL 4, and IL 10, were investigated using qPCR. Our results indicate for the first time that SV2A is also a transporter of understudied phytoflavonoids, due to which a significant improvement was observed in epileptic parameters. The mRNA levels of SV2A were found to be significantly elevated in the PF-treated rats when compared with those of the control rats with epilepsy. Additionally, downregulation of the pro-inflammatory cytokines and upregulation of the anti-inflammatory cytokines were also noted in the PF-treated groups. It is concluded that kaempferol, quercetin, and catechin can effectively decrease the epileptic seizures in our chronic epilepsy rat model to a level that is comparable to the antiepileptic effects induced by levetiracetam drug.

## Introduction

Epilepsy is a neurological disorder that is characterized by recurrent malicious seizures, which are varied in occurrence and severity (Szilágyi et al., 2014). *In vivo* and *in vitro* studies have shown that both flavonoid- and non–flavonoid-containing plants have the potential to improve morbidity in epilepsy (Majeed et al., 2019). Different antiepileptic drugs (AEDs) have been reported with serious side effects along with the development of resistance against the currently available AEDs (Chen et al., 2017; Ramalingam et al., 2013). First-generation AEDs include: carbamazepine, phenobarbital, and valproic acid, while lamotrigine, vigabatrin, tiagabine, topiramate, gabapentin, and levetiracetam (LEV) are classified as second-generation drugs (Ahmad et al., 2017). These drugs exert their antiepileptic effects by modulating ionotropic GABA-A, glutamate receptors, synaptic vesicle 2 A (SV2A) transporters, and ion channels such as Na^+^, Ca^++^, and K^+^ (Noachtar et al., 2008; Leclercq et al., 2020). This eventually results in the fluctuation of the firing properties of neurons (Verrotti et al., 2020). LEV exhibits its antiepileptic effect *via* inhibition of excessive synchronized activity between neurons. Padsevonil (novel antiepileptic drug) has more affinity for SV2A than levetiracetam and brivaracetam. LEV employs its therapeutic effect by binding to SV2A (Patel et al., 2006). LEV is used to treat refractory status epilepticus (Patel et al., 2006), status epilepticus (Rossetti and Bromfield, 2006), and acute and generalized idiopathic myoclonic epilepsy in neonates as well in adults (Khan et al., 2011). LEV has a high affinity for the SV2A receptor, where it binds and modulates the receptor. It has been observed that SV2A does not play a role in biogenesis or synaptic function, but it modulates exocytosis of transmitter‐containing vesicles (Mendoza-Torreblanca et al., 2019). To understand the epileptogenesis and discovery of novel AEDs, chemically kindled epileptic models in rats have been developed. The chemically kindled animals are exposed to sub-conclusive chemical stimuli. When the chemical is applied repetitively and intermittently, it ultimately leads to the generation of complete convulsions (Lynch et al., 2004). PTZ antagonizes the glycine amino butyric acid (GABA)-A receptor that leads to the development of seizures in rats (Hansen et al., 2004; Badawi et al., 2021).

It has been reported that brain inflammation contributes to the etiopathogenesis of seizures and the establishment of chronic epilepsy (Ambrogini et al., 2019). Inflammatory cytokines (IL-1β, TNF-α, and IL-6) and non-inflammatory cytokines (IL1Ra, IL-10, and IL-4) overexpressed and underexpressed, respectively, in experimentally induced seizures at the seizure generation and propagation sites in the brain (Vezzani et al., 2011). Similarly, NFkB, which is a transcription factor, is up-regulated in seizure states. The cytokine receptors are also up-regulated and down-regulated accordingly (Maroso et al., 2010). This type of kindling produces foci in the rat’s hippocampus (Dhir, 2012). There is an association between cytokines and neurotransmitters. The neuronal excitability is cytokine-mediated; hence, it ameliorates the disease conditions (Vezzani et al., 2008). The electrical and chemical kindling models are the most frequently used kindling models. Of these, the most famous model is the chemical kindling model. This model is developed by using sub-conclusive doses of any convulsant chemical, which results in the chronic stimulation of cerebral neurons. Hence, chronic stimulation leads to impulsive seizures (Vrinda et al., 2017). Moreover, electrical kindling can also cause chronic stimulation of neuronal cells by sub-conclusive electrical stimulus, which can cause unprompted generalized convulsions.

Natural products have always been a treatment of interest as an alternative to the available allopathic treatments. In this regard, phytoflavonoids (PFs) have been tested in fully kindled animals. However, studies regarding the clinical pharmacokinetic and pharmacodynamic effects of these flavonoids are scarce. Therefore, studies investigating the active constituents from such medicinal plants as well as their mechanism of action are critical.

In the present study, kaempferol, quercetin, and catechins (phytoflavonoids) are used to identify their potential antiepileptic effect in a rat model. These phytoflavonoids are the constituents of most of the plants that are reported to have antiepileptic potential (Moghbelinejad et al., 2016). These PFs have been shown to be effective against epilepsy, and Alzheimer’s and Parkinson’s diseases (Farooqui and Farooqui, 2012). Catechin protects the brain from producing beta-amyloid protein that results in the deposition of amyloid plaques and memory impairment in Alzheimer’s (Conte et al., 2003), while quercetin’s mechanism of action is unknown; however, it has been shown to be effective in temporal lobe epilepsy (Moghbelinejad et al., 2016). Kaempferol is reported to have antidiuretic and antiarrhythmic potential to cure as well as prevent from counteracting human immune virus 1 (HIV 1) infection (Behbahani et al., 2014).

## Materials and Methods

### Materials

LEV (Batch number L16102017) from Wuhan Demeikai Biotechnology Co., Ltd; quercetin (Batch number HK20181225) from Shaanxi Huike Botanical Development Co., Ltd., P.R. China; kaempferol (Batch number HK20180320) from Shaanxi Huike Botanical Development Co., Ltd., P.R. China; catechin (Batch number 204CELK04) from Sigma-Aldrich, Germany, pentylenetetrazole (PTZ) (Batch number SLBD3876V) from Sigma-Aldrich, Germany; and IR actimeter from Panlab, Harvard apparatus, Barcelona, Spain, were obtained.

In this study, *in silico* evaluation was carried out to explore the possible binding mechanism of PFs with the SV2A receptor. After the *in silico* studies confirmed the binding of PFs with the SV2A receptor, the *in vivo* studies were performed in the chronic epilepsy rat model to establish the proof of concept as well as find confirmation of our *in silico* study. In order to identify the possible mechanism behind the epileptic treatment in our rat model, we also investigated the expression levels of pro- and anti-inflammatory cytokines in the blood of our rat models.

### Study Design

**FIGURE 1:**
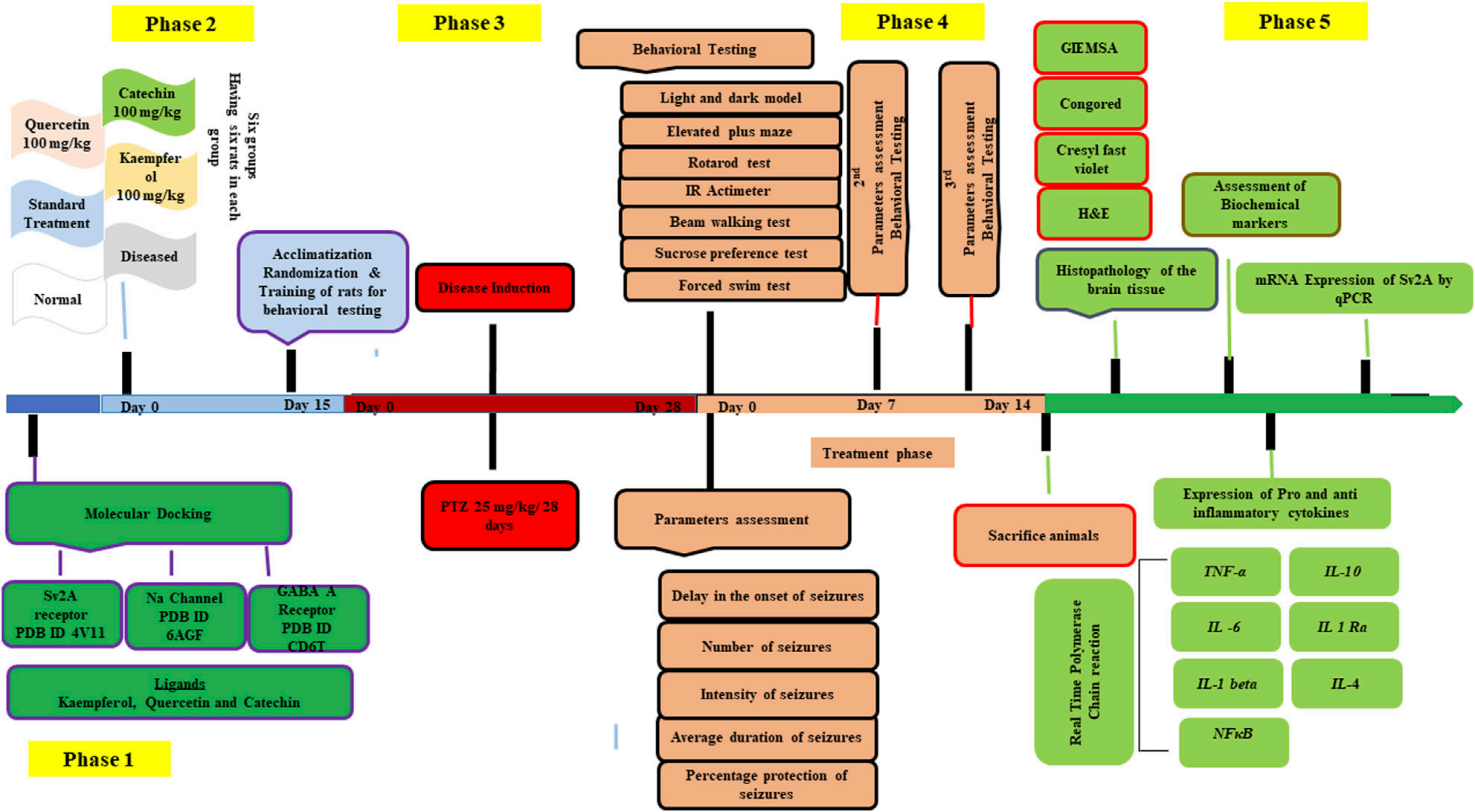
Showing the study design.

Figure 1 outlines the study design.

### *In Silico* Studies

#### Ligand Preparation and Docking Studies

The co-crystal structure of the SV2A receptor (PDB entry: 4V11), sodium channel (PDB entry: 6AGF), and GABA-A (PDB entry: 6D6T) receptors were retrieved from the RCSB Protein Data Bank. After generating the 3D model and structure, stereochemical analyses were performed using different evaluations and validation tools. All the molecules were neutralized by removing water molecules. The Psi/Phi Ramachandran plot was obtained using PROCHECK (Laskowski et al., 1993). The 3D structure of LEV and PFs was constructed using the Sybyl-X1.3/SKETCH module (Jain, 2003)*.*

#### Molecular Dynamic Simulations

The final complexes of LEV and PFs bound to the SV2A receptors were subjected to molecular dynamic (MD) simulations for further stabilization and refinement. All MD simulations were thoroughly performed in PyRx, PyMol, and Autodock software packages (Case et al., 2016).

#### Free Energy Calculation

Binding energies of the selected compound with SV2A complexes were determined. Initially, the equilibrium confirmations for the complex, free receptor, and free ligands were prepared, and after that, the binding free energy was calculated.

### Animals

Male Albino rats (weighing 180–220 g) were purchased from the animal house and were kept under standard laboratory conditions (temperature 25 ± 3°C, humidity 55 ± 5%, and 12 h light and dark cycle). All rats had free access to the standard diet and water.

The rats were randomly divided into six groups (seven rats in each group): normal (Group 1), diseased (Group 2), standard drug (Group 3, LEV 21 mg/kg od), kaempferol (Group 4, 100 mg/kg od), quercetin (Group 5, 100 mg/kg od), and catechin (Group 6, 100 mg/kg od) treated groups. The doses of PFs were chosen based on the literature as the most effective doses to exert the desired pharmacological effects (Diniz et al., 2015).

### Chemical Kindled Model of Epilepsy

The rats were injected intraperitoneally (i.p.) with the sub-conclusive dose of PTZ (25 mg/kg) for 28 days (Yu et al., 2013).

#### Clinical Scoring

The intensity of seizure response was scored according to the scale described as follows (Yao et al., 2012): 0 (no response), 1 (mouth and facial jerks), 2 (nodding or myoclonic body jerks), 3(forelimb clonus), 4 (rearing, falling down, hind limb clonus, and forelimb tonus), and 5 (tonic extension of hind limb or death).

The maximum response was recorded for each animal. Only those animals that maintained a minimum threshold score of 2 in at least five consecutive episodes or a score of 4 or 5 in three consecutive seizure episodes were considered to be the kindled rats (da Silva Ferreira, 2018). The treatment was carried out from day 29 until day 43.

### Parameters

The following parameters were noted during the observation time (360 min after the injection) on days 0, 7, and 14: delay in the onset of seizures (Singh and Goel, 2009), intensity of seizures, average number of seizures, and duration of seizures, while the maximum time frame was up to 360 min, percentage protection of seizures (Garip Ustaoglu et al., 2018).

### Behavioral Tests

The behavioral tests were performed in all groups before and after the induction of epilepsy. Only the animals that met the minimum criteria of the study were considered for behavioral testing. Each animal was housed in an individual plastic cage during the study period. All behavioral tests were performed in a closed, quiet, light-controlled room, and the results were analyzed by an investigator blinded to the treatment.

#### Light and Dark Model

The apparatus consisted of an open-top wooden box with two distinct chambers, one (20 × 30 × 35 cm) darkened with black paint and another (30 × 30 × 35 cm) brightly illuminated with a white light source. Both the chambers were connected through a small open doorway (7.5 × 5 cm) (Gupta and Sharma, 2019). Each rat was placed individually in the center of the light chamber and observed for 5 min. The time spent in the light and dark chambers was recorded (Singh et al., 2019).

#### Elevated Plus Maze

The elevated plus maze (EPM) was made up of a wooden arena, with two opposite arms having 30 cm high walls, while the other two arms were exposed. Each arm has a width of 13 cm and length of 43.5 cm; the maze was elevated 60 cm above the floor.

The training protocol used for the behavioral testing has been reported elsewhere (Tanila et al., 1997). The training was carried to ensure that the animals were previously familiarized with the single-trial procedure and that their choice was reinforced regardless of the arm selected. The training was carried out in the regular morning and afternoon sessions for 15 days.

Each rat was placed in the middle of the EPM, and the total time spent in each arm during the 300 s trial was recorded (Tchekalarova et al., 2014).

#### Rotarod Test

The rotarod apparatus consisted of a roller of 8 cm diameter, separated into wide compartments of 9 cm, and are raised at 16 cm (Oleas et al., 2013). The rats were trained for 3 days: on the first day, over 1 min for four (Chen et al., 2017) times; on the second day, over 1 min for four (Chen et al., 2017) times and 20 rpm; and on the third day, the rotarod accelerated to 40 rpm over 5 min (Jurado-Arjona et al., 2019). The latency to fall was measured during the accelerating trials. The latency to fall was recorded, and the total latencies on the rod on each day were analyzed. Next, to compare the typical rotarod test with our modified rotarod test, we also tested the accelerating rotarod protocol (ref). The speed of the rod was accelerated from 10 to 40 rpm. The habituation time and daily schedule were the same (Shiotsuki et al., 2010).

The rats were positioned on the rod of rotarod and tested with different rotational speeds for 2 min at each speed, beginning at the slowest usually 10 rpm and increasing progressively. The length of time that each animal can remain on the rod at a given rotational speed was observed. The test was performed on several rats in the same session. Usually, after each session, the rat is rested for about 20–30 min, to reduce stress and fatigue (Arcuri et al., 2016).

#### Infrared Actimeter

The IR frame where the experimental subjects were placed had a dimension of 45 × 45 cm. It contains a total of 16 × 16 IR beams at an interval of 2.5 cm located on the sides. There were a total of 32 cells in each frame. The frame is attached with a control unit/data logger, which is manufactured by Panlab, Harvard apparatus, Barcelona, Spain. This apparatus has a unique ability to identify the slow as well as fast movements of the rat placed inside the frame (Yaroslavsky et al., 2019).

The individual rat was placed in the frame for 5 minutes and turned on the data logger (Machado et al., 2012). The data logger counts both slow movements and fast movements of the rat with the help of infrared rays (Busquets et al., 2018). It was found that the diseased rats displayed more slow movements, while the normal healthy rats showed more fast movements comparatively (Studer et al., 2019) (Machado et al., 2012).

#### Beam Walking Test

The beam walking test (BWT) is widely used to evaluate motor coordination and balance in rat models. It consists of a wooden beam (1 m) which is round of shape (5 mm diameter) and elevated 50 cm above the floor. Animals were allowed to reach the closed box (20 × 20 cm) at the end of the beams (Dawes et al., 2018).

The starting point was brightly lit with a 60 W table lamp to produce an aversive motivational stimulus. Initially, the rats were trained for 1 week (7 days) on the beams, limited to 20 seconds (s) to reach the box. On subsequent two attempts, the animals were able to cross the beam in a maximum of 10 s, according to the modified protocol of Carter et al. (2001).

The rats from each group were placed on the beam, and the time they took to cross the beam was determined (Tărlungeanu et al., 2016).

#### Sucrose Preference Test

The sucrose preference test is performed to assess the hedonic behavior in rats. The rats were housed individually to acquaintance with the environment for 24 h. The sucrose solution (1.5% w/v) filled bottle was replaced with one of the two water-filled bottles, to avoid abrupt response. During and after the test, the sucrose volume consumed from each bottle was noted. The sucrose preference was calculated as described earlier (Meyerolbersleben et al., 2020).

#### Forced Swim Test

The FST is one of the most commonly used animal models for assessing locomotory and antidepressant behavior. Active and passive swimming movements are recorded in this test. The rats were forced to swim in a specified vessel with no exit. The container was filled with water up to a level of 30 cm from the bottom at 23–25°C. Reduction in the passive behavior was inferred as an antidepressant-like effect, provided it does not increase the general locomotor activity (Mograbi et al., 2020).

### Histopathology

The five rats from each group were decapitated; the brains were separated immediately and postfixed in 10% V/V formalin solution for 48 h. The longitudinal section of the hippocampus was separated, placed in the tissue cassettes, and fixed in paraffin block for later histopathological analysis. The histopathological analysis was carried out by following the methods (Paradiso et al., 2018).

Different staining methods were carried out to identify the morphological changes in the hippocampal tissue slices, that is, living or dead cells, accumulation of beta-amyloids, and unwanted bodies. Hematoxylin and eosin stain (H&E), Congo red (CR), cresyl fast violet (CFV), and Giemsa stain are some of the staining methods.

### Assessment of Serum Electrolytes

The hematological samples were collected *via* heart puncture. Serum electrolytes and calcium were determined by using serum electrolyte analyzer and assay kit, respectively.

### Expression of SV2A Gene

The whole hippocampus was separated, and total RNA was isolated using TRIzol (Invitrogen). To estimate the mRNA levels of the *SV2A* gene, the primers were designed (Table 1), reverse-transcribed, and standardized as discussed earlier. The ΔΔCT method was used to determine the SV2A mRNA levels in the hippocampus of all animal groups. The data are presented as fold change in the mRNA levels of SV2A (Vanoye-Carlo and Gómez-Lira, 2019).

**TABLE 1:** Forward and reverse primers are tabulated.

Molecule	Forward primer	Reverse primer
Interleukin 1 Beta	5′CCA​CTA​CAA​AAT​CTG​GGC​GAT​GCA3′	5′CAG​AAG​AAG​AGG​ATG​TCA​CTC​CTC​G3′
TNF alpha	5′CAA​AGA​CAA​CCA​ACT​GGT​GG3′	5′TGA​GAT​CCA​TGC​CAT​TGG​CC3′
IL 6	5′CCC​ACC​AAG​AAC​GAT​AGT​CA3′	5′CTC​CGA​CTT​GTG​AAG​TGG​TA3′
NFКB	5′GAG​GCG​TGT​ATT​AGG​GGC​TA3′	5′ACG​CTC​AGG​TCC​ATC​TCC​TT3′
IL-4	5′ACC​GAG​AAC​CCC​AGA​CTT​GT3′	5′GGA​TGT​AAC​GAC​AGC​CCT​CT-3′
IL-10	5′TCT​AGG​CCT​GTA​CGG​AAG​TGT​TAC​T3′	5′AGC​AGT​GCT​GAG​CTG​TGC​AT3′
IL1Ra	5′GGC​CTT​TCT​CAG​AGC​GGA​TGA​AGG3′	5′TCA​CCC​ATG​GCT​TCA​GAG​GCA​GCC3′
GAPDH	5′CTT​GCC​GTG​GGT​AGA​GTC​AT3′	5′TCT​CTG​CTC​CTC​CCT​GTT​CT3′
SV2A	5′CCCAAACATGGGACA3′	5′GAC​CAA​TAA​TTT​CTT​TTA​TCC​CT3′

### Expression of Pro- and Anti-Inflammatory Cytokines

The expression of cytokines was determined within the hippocampus. The hippocampus was separated and homogenized in Trizol solution. The RNA concentration and purity were quantified using a UV microspectrophotometer (NanoDrop). The cDNA was prepared using MagMAX cDNA Kit. The primers were designed using the NCBI database and Primer 3 Plus (Table 1) (El-Missiry et al., 2019).

The RT-PCR conditions were set through the Bio-Rad framework (Sun et al., 2019). GAPDH was used as the housekeeping gene; its sequence is tabulated in Table 1. The ΔΔCT method was used to determine the expression of cytokines and transcription factors.

### Statistical Analysis

The results were analyzed statistically and expressed as mean ± S.E.M. The plots were created using Graph Pad Prism. The Newman–Keuls multiple comparison posttest of two-way analysis of variance (ANOVA) and one-way ANOVA followed by *post hoc* Tukey’s multiple comparisons test, where applicable, was performed to compare the test groups with the control groups. The results were considered as non-significant (ns) if *p* > 0.05, and significant if *p < 0.01. **p < 0.05, ***p < 0.0005, and ****p < 0.0001.

## Results

### *In Silico* Studies

The results were analyzed graphically by visualizing the SV2A protein using PyMol software. The protein backbone is represented in cartoon form. The ligands are shown in ball and stick representation, and the hydrogen bonds are indicated as dashed lines.

**FIGURE 2:**
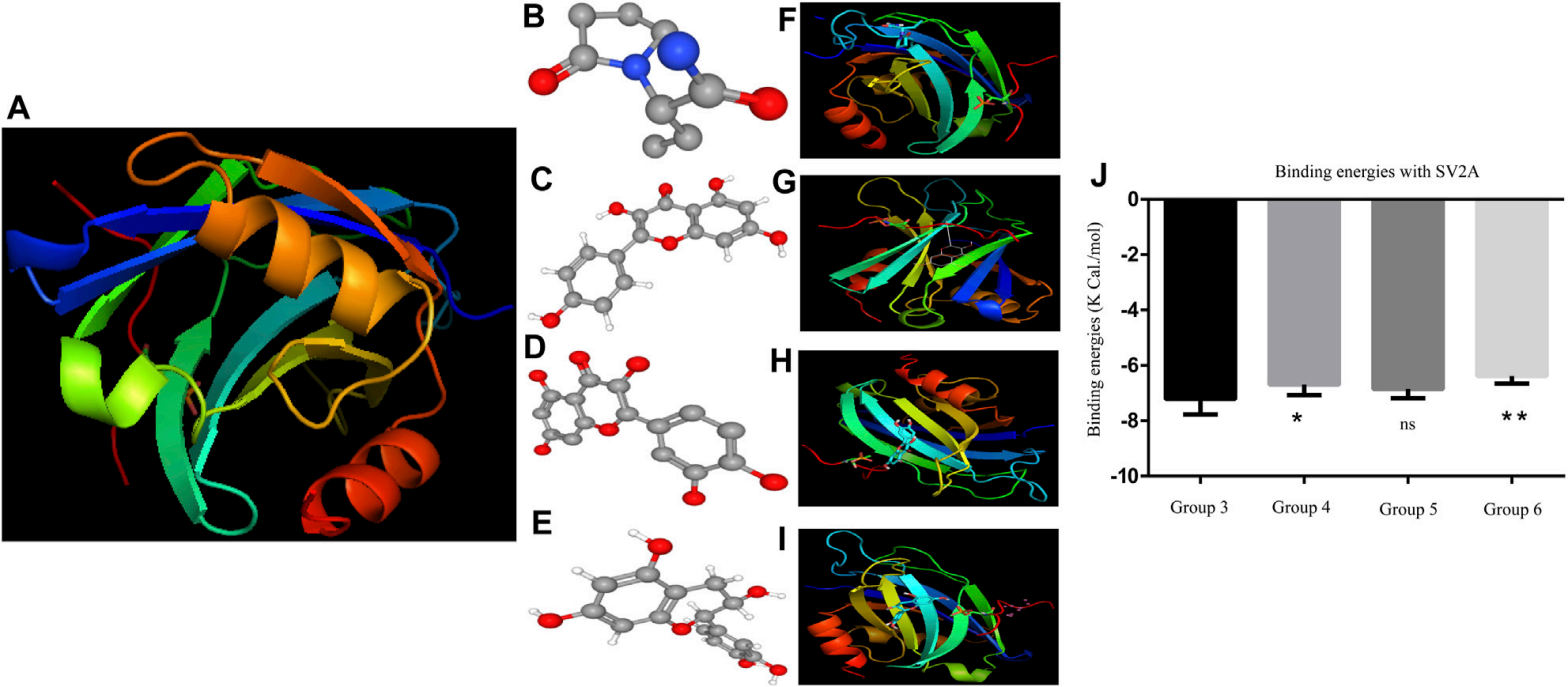
**(A)** SV2A receptor **(B)** is the 3D structure of LEV. **(C–E)** Phytoflavonoids (kaempferol, quercetin, and catechin) **(F–I)** showing the protein ligand interaction of LEV, kaempferol, quercetin, and catechin. **(J)** Graph of the binding energies one-way ANOVA (Moghbelinejad et al., 2016; Verrotti et al., 2020) F = 8.130 followed by *post hoc* Bonferroni’s multiple comparison test.*p < 0.01. **p < 0.05, ***p < 0.0005, and ****p < 0.0001.

Figure 2 represented the best docking pose of the SV2A receptor, while LEV and PFs displayed different binding sites to the receptor. The ligand is docked into the binding cavity of the receptor, and the putative conformations are shown. The docking results show that almost all residues of the compounds are interacting with SV2A receptors, leading to favorable interactions with LEV and PF complexes.

The binding energies of both LEV and the PF bonded system are shown in Figure 2. The binding energies show no significant difference in the binding affinity of quercetin compared to LEV. The free binding energies of kaempferol and catechin were highly significant with *p* < 0.0001 and *p* < 0.001, respectively. PyMol visualization reveals no ligands binding to the 6AGF and 6D6T, with the binding data shown in the supplemental file.

### Delay in the Onset of Seizures

**FIGURE 3:**
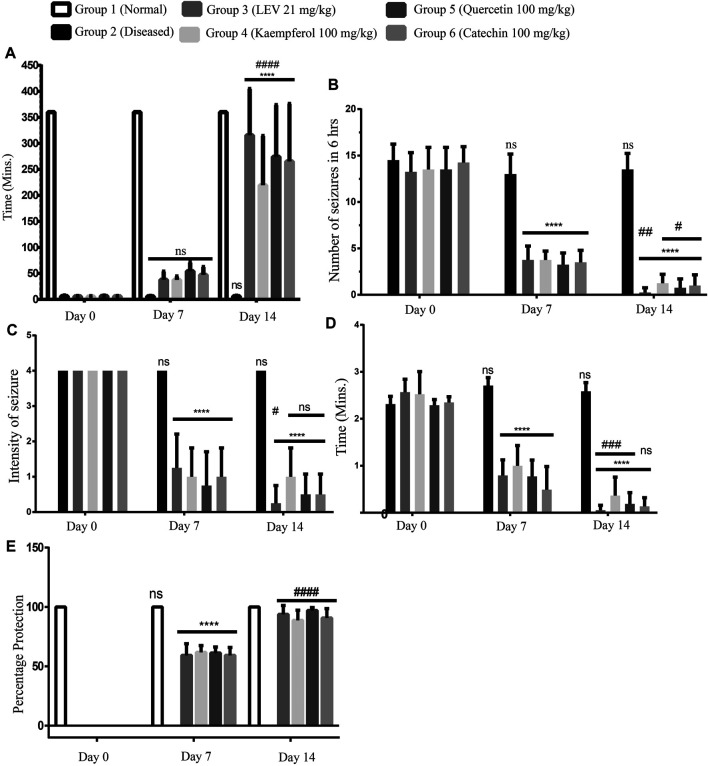
**(A)** Delay in the onset of seizures, Day 0: onset of seizures was less than a minute after induction. At Days 7 and 14 of treatment, onset was delayed in each group up to 60 min and/or up to 360 min (maximum time), respectively. ANOVA, F (5/42) = 15.68, confirmed significant improvement at Day 14 among the groups and also between two time points. While at time points Day 7 and Day 14, there was no significant difference among standard treatment and phytoflavonoid-treated groups. **(B)** Number of seizures, Day 0: number of seizures in all the rats was more than 13; at Day 7 of treatment in the treatment group, there was a significant reduction in the number of seizures. On Day 14, the number of seizures was significantly reduced as compared to both Day 0 and Day 7 until 360 min (6 h). ANOVA, F (5/42) = 88.26, confirmed significance improvement at day 14 among the groups and also between two time points. **(C)** Intensity of seizures, Day 0: Intensity of seizures in all the rats was more than 3; at Day 7 of treatment in the treatment group, there was a significant reduction in the intensity of seizures; at Day 14, the intensity of seizures was significantly reduced as compared to Days 0 and 7 until 360 min (6 h). ANOVA, F (5/42) = 16.25, confirmed significant improvement at Day 14 among the groups and also between two time points. **(D)** Average duration of seizures, Day 0: Average duration of seizures in all the rats was more than 2 min per seizure; at Day 7 of treatment in the treatment group, there was a significant reduction in the duration of seizures; at Day 14, the duration of seizures was significantly reduced as compared to Days 0 and 7 until 360 min (6 h). ANOVA, F (5/42) = 139.4, confirmed significant improvement at Day 14 among the groups and also between two time points. **(E)** Percentage protection of seizure, Day 0: all the groups observed seizures except the normal group. At Days 7 and 14 of treatment, seizures were protected in all the groups, but no protection was observed in the diseased group during the observation time. ANOVA, F (5/42) = 60.30, confirmed significant improvement at Day 14 among the groups and also between two time points. While at time points Day 7 and Day 14, there was a significant improvement among the standard treatment and PF-treated groups. *p* < 0.0001. **p < 0.05, ***p < 0.0005, and ****p < 0.0001 (Newman–Keuls multiple comparison post-test Day 0 vs Day 7 * and Day 7 vs Day 14#).

In a continuous study of 14 days, on Day 0, the seizure episode started in all rats within a minute. After 7 days of treatment, a delay in the onset of seizure was observed in all the treated groups (Groups 3, 4, 5, and 6). On Day 14, most of the treated rats were normalized; that is, no seizures were observed, while in the diseased (Group 2) untreated rats, the seizures continued even after 14 days of disease induction. Among all the treated groups, Group 5 (Quercetin 100 mg/kg) was found to be the most effective in delaying the onset time. The statistical comparison of Day 0 vs Day 7 (ns) and Day 7 vs Day 14 (*p* < 0.0001) revealed that there was a significant time-dependent delay in the onset of seizures in all phytochemical-treated groups as compared to the standard treated drug (Figure 3A).

### Number of Seizures

On day 0, the number of seizures in all the rats was more than 12 (Patel et al., 2006) during the observation time (i.e., 360 min); after the treatment for 7 days with PFs and standard drug, there was a significant decrease in the number of seizures. Figure 3B shows that Group 4 treated with quercetin (100 mg/kg) is most effective in reducing the number of seizures. On day 14, several treated rats became seizure free, and no rat was observed having more than 2 episodes of seizures during the observation time.

### Intensity of Seizures

According to the scoring index (0–5, death at 5), we consider only those rats that have scored 4 (rearing, falling, hind limb clonus, and forelimb tonus) on Day 0. After the treatment for 7 days, there was a significant attenuation in the intensity of the seizures (Figure 3C) in the treated rats. Moreover, treatment for 7 more days showed a significant reduction in the seizure intensity in the standard treated groups, but in all other treated groups, there was a statistically non-significant reduction. But still, there was an improvement in the treatment on Day 14. While in the diseased group, severe intense seizures were observed even on Day 14.

### Average Duration of Seizures

On Day 0, each seizure lasted for more than 2 min. On Day 7, like all other observed parameters, there was also a significant reduction in the duration of the observed seizures. On Day 14, most rats have turned to be seizure free, and the seizure observing rats had very brief episodes of seizures. Among all the treated groups, Group 4 was still found to be very effective in treating the seizures (Figure 3D).

### Percentage Protection of Seizures

On Day 0 in all the rats, the seizures were observed, except in the normal healthy group. The percentage protection of seizures in all the rats was noted. After treating for 7 days, the percentage protection was significantly increased. When comparing the treatment of Day 7 vs Day 14, there was a significant improvement in the protection of seizures. Among all the treated samples, quercetin 100 mg/kg was found to be the most effective (Figure 3E).

### Behavior Tests

#### Light and Dark Model

**FIGURE 4:**
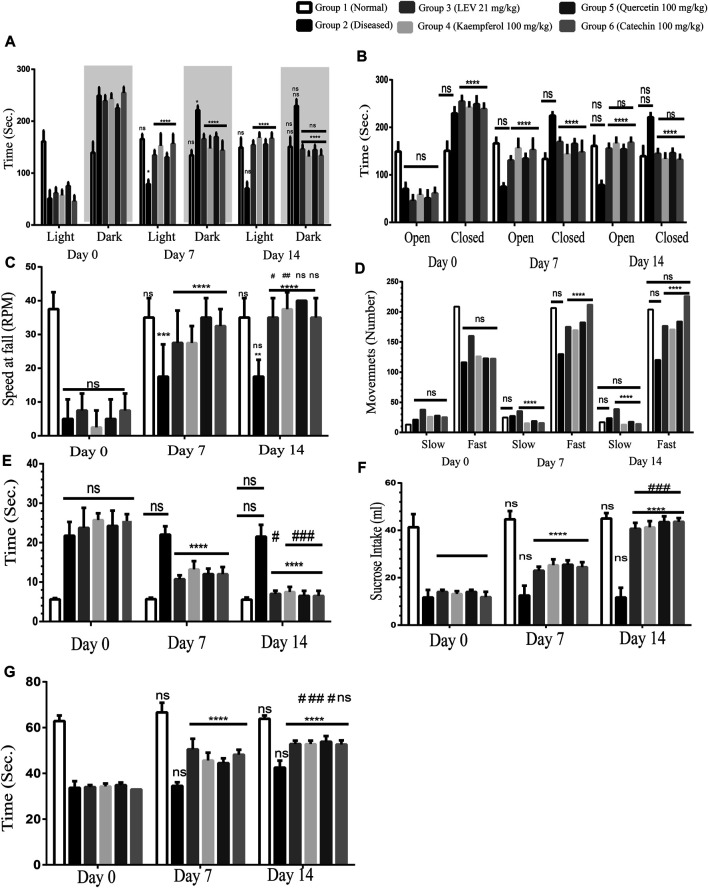
**(A)** Light and dark model, Day 0: diseased rats were non-significant in all the groups showing the proper induction of the disease in all the rats. 7- and 14-Day treatment in both standard treated and others; phytochemicals (100 mg/kg) significantly reduced the time spent in the darker area, and more time is spent in the light area. ANOVA, F (5/42) = 2.33, confirmed significant improvement in the behavior at Day 14 among the groups and also between two time points (Day 7 vs Day 14^#^) was found to be statistically non-significant. **(B)** Elevated plus maze, Day 0: diseased rats were non-significant in all the groups showing the proper induction of the disease in all the rats. 7- and 14-Day treatment in both standard treated and others; phytochemicals (100 mg/kg) significantly reduced the time spent in the closed arm, and more time is spent in the open arm. ANOVA, F (5/42) = 2.33, confirmed significant improvement in the behavior at Day 14 among the groups and also between two time points. Day 7 vs Day 14 was found to be statistically non-significant. **(C)** Rotarod test, Day 0: diseased rats were non-significant in all the groups showing the proper induction of the disease in all the rats. 7- and 14-Day treatment in both standard treated and others; phytochemicals (100 mg/kg) significantly improved the coordination of the rats on the rotarod. ANOVA, F (5/42) = 39.34, confirmed significant improvement in the behavior at Day 14 among the groups. Day 7 vs Day 14 was found to be statistically non-significant. **(D)** Infrared actimeter, Day 0: diseased rats were non-significant in all the groups showing the proper induction of the disease in all the rats. 7- and 14-Day treatment in both standard treated and others; phytochemicals (100 mg/kg) significantly improved the locomotion of the rats on the IR actimeter. ANOVA, F (5/42) = 54.27, confirmed significant improvement in the behavior at Day 14 among the groups. Day 7 vs Day 14 was found to be statistically non-significant. **(E)** Beam walking test, Day 0: diseased rats were non-significant in all the groups showing the proper induction of the disease in all the rats. 7- and 14-Day treatment in both standard treated and others; phytochemicals (100 mg/kg) significantly improved the limb coordination of the rats on the walking rod. ANOVA, F (5/42) = 139.4, confirmed significant improvement in the behavior at Day 14 among the groups. Day 7 vs Day 14 was found to be statistically non-significant. **(F)** Sucrose preference test, Day 0: diseased rats were non-significant in all the groups showing the proper induction of the disease in all the rats. 7- and 14-Day treatment in both standard treated and others; phytochemicals (100 mg/kg) significantly enhanced sucrose containing water. ANOVA, F (5/42) = 7.58, confirmed significant improvement in the behavior at Day 14 among the groups and also between two time points (Day 7 vs Day 14^#^) was found to be statistically less significant. **(G)** Forced swim test, Day 0: diseased rats were non-significant in all the groups showing the proper induction of the disease in all the rats. 7- and 14-Day treatment in both standard treated and others phytochemicals (100 mg/kg) significantly increase the swimming time. Two-way ANOVA, F (5/42) = 12.45, confirmed significant improvement in the behavior at Day 14 among the groups and also between two time points. *p* < 0.0001. *p < 0.01, **p < 0.05, ***p < 0.0005, and ****p < 0.0001 (Newman–Keuls multiple comparison post-test Day 0 vs Day 7 * and Day 0 vs Day 14^*^).

It was observed that on Day 0 all the rats were diseased; therefore, they were spending more time in the dark area than in the light area. This depressed behavior of the rats indicated the proper induction of the disease. After the treatment for 7 days, the diseased group was displaying the same behavior, while in Group 3 (standard treated group), the rats were spending more time in the light area than in the dark area. A similar effect was also observed in Groups 4, 5, and 6 (PF 100 mg/kg) treated groups. After the treatment for 14 days, the diseased rats were still spending more time in the darker areas, whereas Group 3 and Groups 4,5, and 6 (PF 100 mg/kg) treated rats spent significantly less time in the dark area than in the light area (Figure 4A). Among the other treated groups, Group 4 is found to be most effective on Day 7. On Day 14, the treatment was not statistically significant as compared to that on Day 7, while the treatment was highly significant as compared to the diseased group, that is, Day 0. Henceforth, quercetin 100 mg/kg treated group 4 displayed better behavior than the standard treated groups.

#### Elevated Plus Maze

On Day 0, all the diseased rats were spending non-significantly more time in the closed arm, confirming the disease is uniformly induced in all the rats except Group 1 (normal healthy rats). On Day 7, Group 3 standard drug (LEV 21 mg/kg) rats were significantly spending more time in the open arm, and the PF-treated groups were also behaving identically. In comparison between Days 0 and 14, there was also a significant improvement in all the treatment groups. However, by comparing Day 7 vs Day 14, statistically non-significant differences in behavior were observed along with an increase in the open arm stay. Among all the PF-treated groups, quercetin (100 mg/kg) was found to be the most effective (Figure 4B).

#### Rotarod Test

This test was performed to recognize motor coordination in rats. Normal rats remained at the rotating rod up to the speed of 40 revolutions per minute (RPM). Stay time at the rod was more than 3 min. However, the diseased rats were not capable to maintain balance on the rotating rod at a speed of 20 rpm. On Day 0, all the groups were statistically significant, confirming that the disease has been induced, while the normal rats still showed good balance. After 7 days of treatment, there was an incredible improvement in the motor coordination of treated animals in both the standard and PF-treated groups (Groups 3, 4, 5, and 6). Both the treated groups displayed a very significant reinforcement in the normal behavior. Similarly, on Day 14 as compared to Day 0, the rats were showing a significant improvement in the symptoms of epilepsy. In comparing Day 7 vs Day 14, although the improvement in the behavior was found, the results were not significant. Quercetin 100 mg/kg was found to be the most effective in the reinforcement of normal healthy behavior (Figure 4C).

#### Infrared Actimeter

In this test, we quantified the slow movements as well as the fast movements. Normal rats showed normal movements (Figure 4D), while in the diseased group, the slow movements were increased, and fast movements were decreased as compared to those of the normal rats. After the treatment for 7 days in the standard (LEV 21 mg/kg) treated groups, a significant increase in fast movements and decrease in slow movements was observed compared to the normal rats. However, the PF (100 mg/kg) treated groups were significantly the best. The catechin 100 mg/kg treated group showed better results among all the PFs.

#### Beam Walking Test

In the diseased rats, there was a significant delay in passing the rod as compared to that of the normal rats. After the treatment for 7 days in the standard treated group (LEV 21 mg/kg), there was a significant delay in the time to pass the beam. However, in the PF (100 mg/kg) treated groups, there was a very significant (*p* < 0.0001) reinforcement of the normal limb coordination. After the treatment for 14 days, there was a significant (*p* < 0.0001) improvement in normalizing the epileptic symptoms. By comparing Day 7 with Day 14, a significant improvement was found in the gait-related behavior in the standard and PF-treated groups with *p* < 0.01 and *p* < 0.0005, respectively. The catechin 100 mg/kg treated group behaved well among all the treated groups (Figure 4E).

#### Sucrose Preference Test

The diseased rats displayed diminished sucrose preferences. On Day 0, the disease was induced uniformly, except the normal group. Over the course of 7 days of treatment, the standard treated and PF-treated rats showed significant improvement in the sucrose preference. Upon further treatment of 7 days, there was a significant improvement in the sucrose preference. In comparing Day 0 vs Day 7 and Day 0 vs Day 14 (*p* < 0.0001), there was a highly significant improvement, while the normal control and diseased groups remain non-significant. However, by comparing Day 7 vs Day 14, a significant improvement was also observed (*p* < 0.005) (Figure 4F).

#### Forced Swim Test

The swim test involves the monitoring of active mobility and drowning of rats. It was observed that on Day 0, all the rats started drowning after less than 40 s, while normal rats kept swimming for more than 60 s. After the treatment of 7 days with standard drugs and PFs, there was a significant improvement in swimming. Comparing Day 0 vs Day 7 and Day 0 vs Day 14, a highly significant improvement was observed, while the normal control and diseased groups remained non-significant, as shown in Figure 4G.

### Staining

#### Hematoxylin and Eosin

**FIGURE 5:**
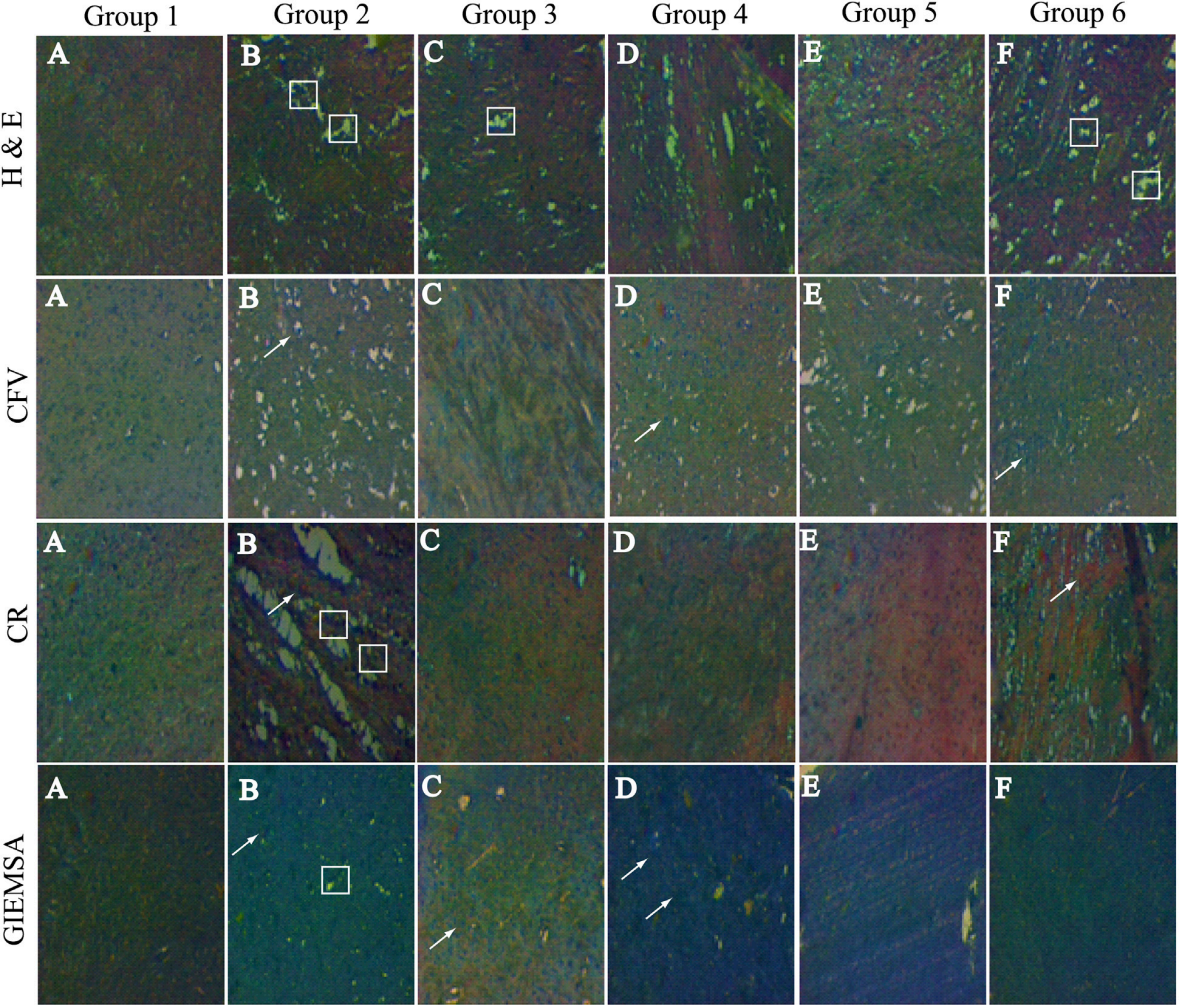
Hematoxylin and eosin staining: Group 1 is showing normal cells in Groups 2, 3, 4, 5, and 6 and displaying the damaged cells. Cresyl fast violet (CFV) staining: Group 1 Nissl bodies are not seen; however, in Group 2, the Nissl bodies ( ) are highly prominent. In group 3 these bodies are not seen, in PF treated groups very few Nissl bodies are stained. Congo red staining CR: Group 1 neuropils and amyloid bodies are not stained, while in Group 2, neuropils and amyloid bodies ( ) are seen. Neuropils are not seen in any other group; however, F amyloid bodies are stained. Giemsa staining: Group 1 glial cells ( ) are visible. In Group 2, glial cells are not visible, and it contains neuropils. In Groups 2,3,4,5, and 6, glial cells are undamaged.

Showing the hippocampal cross-sectional view of the brain tissue, the hematoxylin stains cell nuclei blue, and eosin stains the extracellular matrix and cytoplasm pink. Group 1 is showing the intact cellular morphology, while there is significant damage destruction of the cells seen in Group 2. However, treated groups (Groups 3, 4, 5, and 6) are showing the prevention of cellular destruction among the different groups. Group 5 is showing the normal cellular density (Figure 5).

#### Cresyl Fast Violet

CFV stained the Nissl proteins. The Nissl proteins are displayed in pink to violet color. Group 2 is presented with extreme peripheral and mild centrally stained proteins. The observed intense chromatolytic change in the hippocampal cells was prevented by the treatment in the treated groups (Figure 5).

#### Congo Red Staining

The CR-stained hippocampal cells are shown in Figure 3. The stained sections of hippocampal cells exhibited dull red staining of the neurons as well as the neuropil. Congophilic masses of irregular sizes were seen. Amyloid deposits are visible as red to pink-red, the nucleus is blue, and all other tissues did not stain and can be seen by an ordinary light microscope. Inflammatory cells and granular basophilic debris were stained dark blue to black. Group 2 is showing both dull and bright red neuropil and amyloid proteins, respectively. While in the treated groups (Groups 3, 4, 5, and 6), the chromolytic changes were not observed (Figure 5).

#### Giemsa Staining

The neurons appear red-violet, while cerebellar granules are seen as deep blue; however, the glial cells are visible in pale blue. At high magnification, the nucleus of glial cells is stained blue and scarce cytoplasm red-violet. The astrocyte nuclei are stained homogeneously, while the neuropil is seen as bright pink, giving an enhancing framework for the cells. Non-myelinated and myelinated fibers will not be stained.

Group 1 displays glial cells having normal nuclei, cytoplasm, and astrocytes include normal neuropils. However, group 2 is displaying the ruptured nucleated cell. Likewise, the treated groups are showing lesser damage to the hippocampal cells than the diseased group (Figure 5).

### Assessment of Sodium, Potassium, Chloride, and Calcium Levels

The results showed significant fluctuations in the diseased groups; however, treatment with PFs regularizes the levels of these electrolytes as also shown by the standard treated group (Table 2).

**TABLE 2:** Levels of sodium potassium chloride and calcium levels: Group A (normal control), Group B (PTZ 25 mg/kg), Group C (LEV 21 mg/kg), Group D (kaempferol 21 mg/kg), Group E (quercetin 100 mg/kg), and Group F (catechin 100 mg/kg). Statistical analysis was done through one-way analysis of variance (ANOVA) trailed by Tukey’s *post hoc* test for all groups. The results are considered significant ^∗^ if *p* < 0.005. ^∗∗^ *p* < 0.05 and ^∗∗∗^ *p* < 0.05. Results were compared in a column with the respective diseased groups.

Groups	Sodium (mEq/mmol)	Potassium (mEq/mmol)	Chloride (mEq/mmol)	Calcium (mEq/mmol)
A	135 ± 5.5**	4.0 ± 1.25**	105 ± 2.2*	12 ± 1.5**
B	155 ± 5	9.6 ± 0.8	110 ± 2.5	6 ± 0.5
C	135 ± 2**	5.0 ± 0.5*	104 ± 3*	11 ± 0.5**
D	140 ± 5*	6.4 ± 0.2*	105 ± 4*	11 ± 1.0**
E	142 ± 3*	5.2 ± 0.25*	106 ± 2ns	12 ± 0.5***
F	138 ± 4*	5.8 ± 0.84*	108 ± 5ns	11 ± 0.5**

### mRNA Expression of SV2A

**FIGURE 6:**
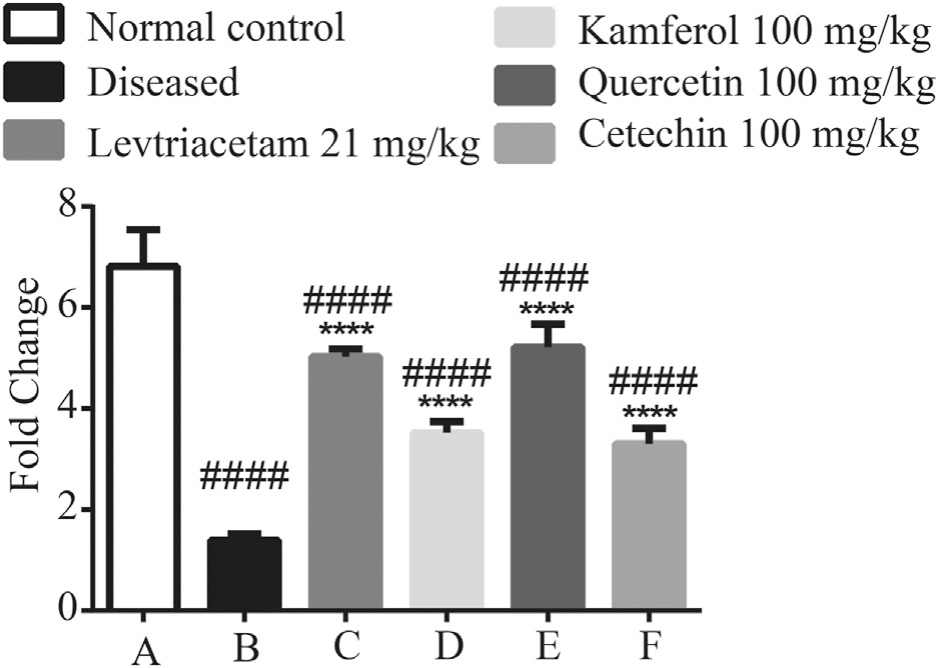
Graphical representation of the mean ± SD relative expression levels of SV2A. The treatment of PF significantly improves the protein expression. The data were analyzed by one-way ANOVA followed by Tukey’s *post hoc* test. Comparison of Group A vs B, C, D, E, and F denoted by (#), while Group B vs C, D, E, and F is denoted by (*). *p < 0.05. **p < 0.01, ***p < 0.0005, and ****p < 0.0001.

SV2A is highly expressed in hippocampal tissue in the diseased states. The expression of the receptors was significantly reduced. After the treatment for 14 days, standard drug and PFs showed a significant amplification in the RNA (Figure 6).

### mRNA Expression of Pro-Inflammatory Cytokines

**FIGURE 7:**
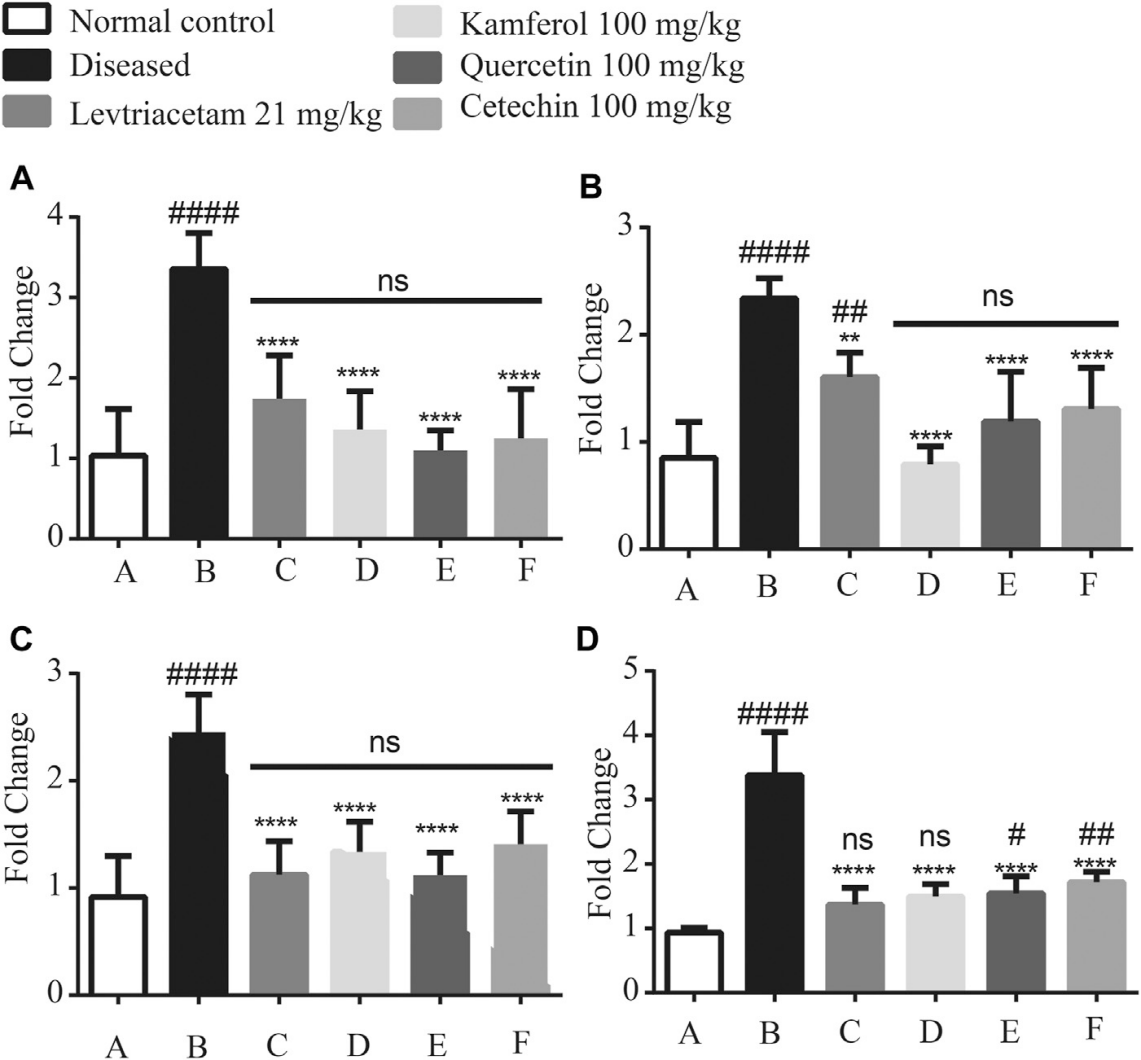
Graphical representation of the mean ± SD relative expression levels of **(A)** TNF-α, **(B)** IL-6, **(C)** IL1 beta, and **(D)** NFkB. The treatment of PF significantly suppresses the pro-inflammatory cytokines. The data were analyzed by one-way ANOVA followed by Tukey’s *post hoc* test. Comparison of Group A vs B, C, D, E, and F denoted by (#), while Group B vs C, D, E, and F is denoted by (*). *p < 0.05. **p < 0.01, ***p < 0.0005, and ****p < 0.0001.

Expression levels of pro-inflammatory cytokines such as TNF-α, IL-6, IL-1 beta, and NF-КB were significantly up-regulated in diseased group B as compared to that of normal group A. The treatment with LEV and PF groups (C, D, E, and F), respectively, caused a significant reduction in the expression levels. Similarly, compared with Group A, it revealed that non-significant difference was observed in Groups C, D, E, and F (Figure 7).

### Expression of Anti-Inflammatory Cytokines

Anti-inflammatory cytokines including IL-10, IL-1Ra, and IL-4 levels were found significantly down-regulated in diseased group B as compared to that of normal group A (44.49 ± 6.39). These reduced levels were found up-regulated after the treatment with LEV and PFs.

**FIGURE 8:**
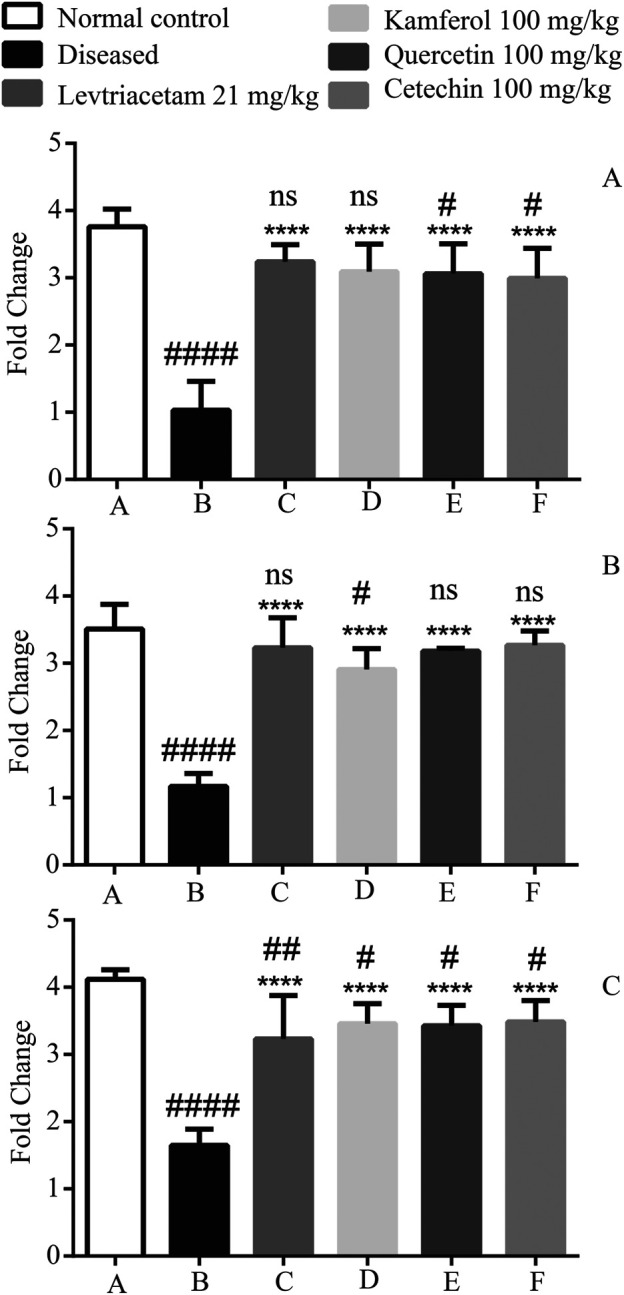
Graphical representation of the mean ± SD relative expression levels of **(A)** IL-1Ra, **(B)** IL-4, and **(C)** IL-10. The treatment of PF significantly suppresses the pro-inflammatory cytokines. The data were analyzed by one-way ANOVA followed by Tukey’s *post hoc* test. Comparison of Group A vs B, C, D, E, and F denoted by (#), while Group B vs C, D, E, and F is denoted by (*). *p < 0.05. **p < 0.01, ***p < 0.0005, and ****p < 0.0001.

By comparing normal group A with treated groups (C, D, E, and F), IL-10 groups D, E, and F had *p* < 0.01 and C had *p* < 0.05 levels significant. In case of cytokine IL-1Ra Groups C and D were non-significant, while in Groups E and F (*p* < 0.05) significance was observed likewise in IL-4 Group D (*p* < 0.05) significance was observed, and the rest of the treated groups were non-significant (Figure 8).

## Discussion

Epileptic seizures are triggered by the imbalance between the activity of excitatory and inhibitory neurotransmitters (Higuera-Matas et al., 2012). The etiology of seizures is unknown; however, certain stimuli can provoke, initiate, or augment the number, duration, frequency, and intensity of seizures (Ancelin et al., 2007). Other pathogenetic factors include the up-regulation of the pro-inflammatory cytokines (TNF α, interleukin (IL)-1β, and IL-6) and down-regulation of the anti-inflammatory cytokines (IL-1 receptor antagonist, IL-4, and IL-10) in the hippocampal tissues. Likewise, up-regulation of the transcription factor NFkB has also been observed (Šutulović et al., 2018).

Molecular docking of both LEV and PFs to the active site of the SV2A receptor was performed. Docking poses for both proteins were saved for each complex and graphically viewed to identify the ligand–protein interactions. Both ligands developed similar types of interactions at the dynamic site of the protein; at this site, it formed similar H-bond interactions with adjacent residues (Jain, 2003). The docking results showed the involvement of receptor residues, that is, Lys 333, Ala 396, and Leu 281, in docking all the ligands. This elucidates that the binding sites on SV2A for LEV and PFs are apparently similar to each other, which further signifies that both ligands probably possess the same therapeutic efficacy. Although molecular docking investigations only outline the binding of ligands with the protein, the solvent, temperature, and pressure effects could not be considered (Fogolari et al., 2003). Moreover, it outlines the possible drug targeting sites on this protein as well as their role in stabilizing ligand–protein interactions. Further research on the pharmacokinetics and pharmacodynamics of PFs will help to underpin the downstream signaling pathways.

PTZ is used to develop chronic chemically kindled epilepsy models. PTZ is GABA-A receptor antagonist. It is a tool to study hippocampal foci–induced epilepsy and its effects, by altering the GABA and glutamate concentration. SV2A is concentrated in the hippocampus. In the principal cell layers (granular and pyramidal), the SV2A protein is localized to GABAergic and glutamatergic terminals. SV2A is ubiquitously expressed in the entire hippocampus; it established an association with excitatory or inhibitory terminals, and this could lead to maintaining the balance of excitatory/inhibitory neurotransmitters. SV2A belongs to a unique family of proteins that is exclusive to secretory vesicles that endure calcium exocytosis (Ciruelas et al., 2019).

Due to the excessive neuronal firing, generalized seizures were observed in the rats. Oxidative stress is an important mechanism in several neurological disorders (Pearson-Smith and Patel, 2017). Oxidative stress is experienced after the first episode of seizure, which then leads to epileptogenesis (Pearson-Smith et al., 2017).

Unlike conventionally used AEDs, LEV did not show effectiveness in the primitively employed acute epilepsy animal models. However, it showed effectiveness in the chronically kindled epilepsy model. The SV2A binding affinity of LEV and its derivatives correlated strongly with their binding affinity in the brain, as well as with their ability to protect against seizures. The specific effect of LEV binding to SV2A proves that there is a reduction in the rate of vesicle release (Yang et al., 2007). Flavonoids may cause the facilitation of the GABAergic and glutamatergic neurotransmission. Flavonoids are antioxidants and can cross the blood–brain barrier (ref). Quercetin has been shown to antagonize GABA-A receptors (Nassiri-Asl et al., 2016). Catechin could enhance the spatial cognitive ability in normal animals. Recent reports demonstrate that catechin could attenuate oxidative damage in the brain ischemia model. These properties enhance the probability of catechin as a substance used in treating epileptic seizures (Wang et al., 2016).

On Day 0, all the rats were completely kindled as shown in the results of the observed parameters and verified by the behavior tests as compared to Group 1 (normal rats). After the treatment for 7 days, PFs displayed comparable results with Group 3 (LEV treated). Furthermore, treatment was continued for 7 more days, that is, up to 14 days. Kaempferol (Group 4) and quercetin (Group 5) treated groups demonstrated better results than LEV (Group 3) treated groups. In all the treated groups, the onset time, intensity, number, average duration of seizure, and percentage protection of seizures were ameliorated in comparison to LEV.

PTZ-induced seizures are also characterized as impairment in spatial learning ability, reference memory, coordination, and locomotion in the rat model (ref). The hippocampus plays its role in learning and memory, especially in spatial cognitive learning, locomotion, and coordination (Malone et al., 2008). Changes in the synapses have a direct impact on the performance of rats in rotarod, IR actimeter, beam walking test, elevated plus maze, and light and dark model. After 14 days of treatment, all the rats were behaving like normal rats. Epilepsy prompts anxiety and depression (Aguilar et al., 2018). This behavior was evident in the diseased rats. Furthermore, elevated plus maze and light and dark models were used to recognize the anxiety and depression in the rat model. Similar to LEV, these PFs have the potential to decrease epilepsy-induced depression and anxiety. A markedly reduced interest or pleasure in activities previously considered pleasurable is one of the main symptoms in neurodegenerative diseases (ref). The sucrose preference test has explicitly highlighted the cognitive system in sucrose preference. This behavior was evident in the diseased rats. These tests are used to identify the expression of neurodegeneration due to the disease. Furthermore, these tests were tested to recognize anxiety and depression symptoms due to chronic epileptic seizures. Similar to LEV, these PFs have the potential to decrease epilepsy-induced depression and anxiety.

Histopathological analysis of the brain tissues revealed that these flavonoids have the potential to decrease or prevent cellular damage. These PFs are antioxidants in nature. Besides that, CFV stained the Nissl bodies in the brain. These bodies were formed in neuronal cytoplasm as a result of neuronal inflammation. The bodies were seen in Group 2 (diseased), whereas these bodies were not seen in the treated groups. It is apparent that there was a significant reduction in neuronal inflammation. CR stain confirms the amyloidosis; the amyloid bodies were visible in Group 2 (diseased). However, these were not visible in the treated and normal groups. In this regard, LEV has been shown to be effective against Alzheimer's pathology that is characterized by the accumulation of the beta-amyloid in the neurons (Inaba et al., 2019). This unique drug also possesses anti-inflammatory effects (Gulcebi et al., 2018). Giemsa-stained cells displayed the neurons, glial cells, and astrocytes. The diseased group confirms the presence of damaged neurons, the potential flavonoid diminution of the neuronal damage. It has been observed by the molecular docking results that these flavonoids have a binding affinity toward the SV2A receptor.

The seizure-induced brain damage causes cerebral inflammation, and it initiates the epileptogenic effects (Stienen et al., 2011). Pro- and anti-inflammatory cytokines are highly expressed in the hippocampus. The hippocampal foci were formed in the epileptic rats because of PTZ-induced epilepsy. The expression of these cytokines was accessed in the hippocampal tissue. PTZ formed the epileptic foci in the hippocampus. The hippocampal lesions cause the up-regulation and down-regulation of pro-inflammatory cytokines (TNF-α, IL-1 beta, and IL-6), and anti-inflammatory cytokine (IL1Ra, IL-4, and IL-10) expression, respectively. Pro-inflammatory cytokines stimulate the chronic release of excitable neurotransmitters and inhibit the uptake of these neurotransmitters (Al-Shorbagy et al., 2013). The LEV and phytoflavonoids down- and upregulate the pro-inflammatory and anti-inflammatory cytokines, respectively. We also assessed the expression of the *SV2A* gene. This receptor is most concentrated in the hippocampal regions. In the diseased conditions, the *SV2A* gene is underexpressed (Vanoye-Carlo and Gómez-Lira, 2019). PFs and LEV significantly enhanced the *SV2A* gene expression. Like LEV, PF not only regulates the cytokines but also controls the expression of the receptor. Extensive *in vitro* studies including electroencephalography (EEG) and clinical studies are required to establish the role of these PFs in epilepsy.

## Conclusion

*In silico* studies prove that these PFs have the binding potential toward the SV2A receptor. *In vivo* findings showed that these PFs have the potential to modulate the immune response and can be an important therapeutic alternative for the treatment of epilepsy.

## Data Availability

The original contributions presented in the study are included in the article/Supplementary Material, and further inquiries can be directed to the corresponding author.

## Abbreviations

AEDs, antiepileptic drugs; CFV, cresyl fast violet; CR, congo red; EPM, elevated plus maze; GABA, glycine amino butyric acid; H &E, hematoxylin and eosin; IR, infrared; LEV, levetiracetam; MD, molecular dynamics; PFs, understudied phytoflavonoids; PTZ, pentylenetetrazole; RPM, revolutions per minute; SV2A, synaptic vesicle 2A.
