# Supplementary material for: In Silico and In Vivo: Evaluating the Therapeutic Potential of Kaempferol, Quercetin, and Catechin to Treat Chronic Epilepsy in a Rat Model
Source: Front Bioeng Biotechnol. 2021 Nov 4;9:754952. doi: 10.3389/fbioe.2021.754952 (PMC8599161; doi:10.3389/fbioe.2021.754952)
Supplement: Supplementary file 1 [file DataSheet1.docx]

**Docking of Kaempferol, Quercetin and catechin with Na channel**

The results shows that these ligands could not interact with sodium channel as shown in figures A.

C

B

A


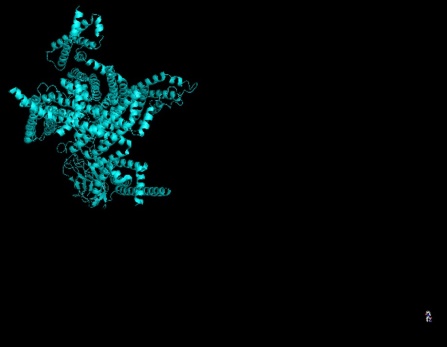

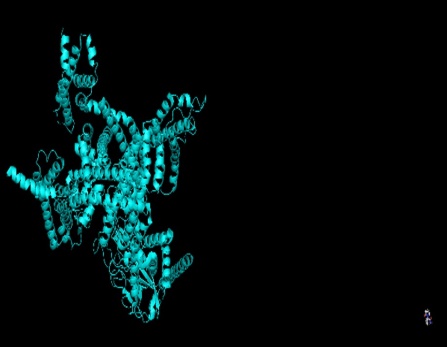

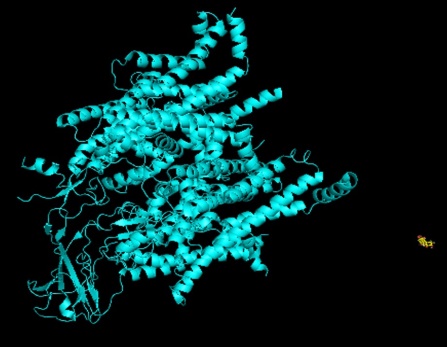


Figure 1 Showing the non-interacting ligands (A) Kaempferol, (B)Quercetin (C) Catechin with Sodium channel (PDB ID: 6AGF)

**Docking of Kaempferol, Quercetin and catechin with GABA-A**

The results showed that these ligands could not interact with GABA-A receptor as shown in figures B.

C

B

A


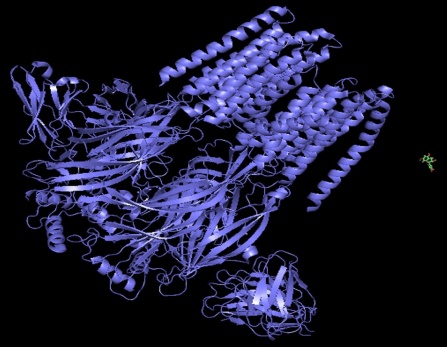

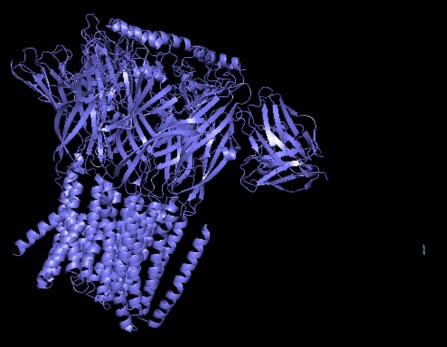

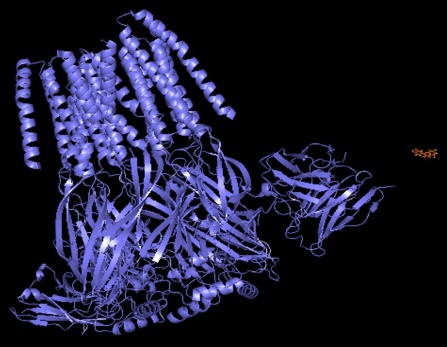


Figure 2 Showing the non-interacting ligands (A) Kaempferol, (B)Quercetin (C) Catechin with GABA A receptor (PDB ID: 6D6T)
